# Contribution to a reference library of DNA barcodes of Colombian freshwater fishes

**DOI:** 10.3897/BDJ.10.e65981

**Published:** 2022-01-06

**Authors:** Manuela Mejía-Estrada, Luz Fernanda Jiménez-Segura, Marcela Hernández-Zapata, Iván D Soto Calderón

**Affiliations:** 1 Grupo de Ictiología, Instituto de Biología, Universidad de Antioquia, Medellín, Colombia Grupo de Ictiología, Instituto de Biología, Universidad de Antioquia Medellín Colombia; 2 Laboratorio de Genética Animal, Grupo de Investigación en Agrociencias, Biodiversidad y Territorio, Instituto de Biología, Universidad de Antioquia, Medellín, Colombia Laboratorio de Genética Animal, Grupo de Investigación en Agrociencias, Biodiversidad y Territorio, Instituto de Biología, Universidad de Antioquia Medellín Colombia

**Keywords:** Cytochrome c oxidase subunit I (COX1), DNA barcode, exotic species, ichthyofauna, ichthyology, occurrence records

## Abstract

**Background:**

The Barcode of Life initiative was originally motivated by the large number of species, taxonomic difficulties and the limited number of expert taxonomists. Colombia has 1,610 freshwater fish species and comprises the second largest diversity of this group in the world. As genetic information continues to be limited, we constructed a reference collection of DNA sequences of Colombian freshwater fishes deposited in the Ichthyology Collection of the University of Antioquia (CIUA), thus joining the multiple efforts that have been made in the country to contribute to the knowledge of genetic diversity in order to strengthen the inventories of biological collections and facilitate the solution of taxonomic issues in the future.

**New information:**

This study contributes to the knowledge on the DNA barcodes and occurrence records of 96 species of Colombian freshwater fishes. Fifty-seven of the species represented in this dataset were already available in the Barcode Of Life Data System (BOLD System), while 39 correspond to new species to the BOLD System. Forty-nine specimens were collected in the Atrato River Basin and 708 in the Magdalena-Cauca asin during the period 2010-2020. Two species (*Loricariichthysbrunneus* (Hancock, 1828) and *Poeciliasphenops* Valenciennes, 1846) are considered exotic to the Atrato, Cauca and Magdalena Basins and four species (*Oncorhynchusmykiss* (Walbaum, 1792), *Oreochromisniloticus* (Linnaeus, 1758), *Parachromisfriedrichsthalii* (Heckel, 1840) and *Xiphophorushelleri* Heckel, 1848) are exotic to the Colombian hydrogeographic regions. All specimens are deposited in CIUA and have their DNA barcodes made publicly available in the BOLD online database. The geographical distribution dataset can be freely accessed through the Global Biodiversity Information Facility (GBIF).

## Introduction

Neotropical freshwater fishes constitute the most diverse continental vertebrate fauna on Earth, with more than 6,200 nominal species concentrated in less than 0.5% of the total land surface, representing the greatest phenotypic disparity and functional diversity of any continental ichthyofauna (Albert et al. 2020). This fauna is still in a pioneering stage of discovery, with dozens of new species being described each year. The current pace of discovery indicates the actual diversity of Neotropical freshwater fishes could exceed 9,000 species, meaning that as many as one-third of the species in the wild still remain to be described (Reis et al. 2016).

Colombia is the second most diverse country in terms of freshwater fishes, comprising 1,610 species (DoNascimiento et al. 2018). In particular, the trans-Andean Basin of the Magdalena and Cauca Rivers exhibits altitudinal gradients, as well as geological and climatic events that have favoured the emergence of a wide diversity of fishes. A total of 232 fish species have been registered in this Basin, of which 57% are endemic (DoNascimiento et al. 2018). The Atrato River Basin, in the Pacific hydrogeographic region, has 128 described species, 32 endemic to this region (DoNascimiento et al. 2018). However, despite the efforts of multiple organisations to collect data, much remains to be known about the diversity of freshwater fishes in Colombia, where the high diversity of fishes, the shortage of specialised taxonomists and taxonomic issues are the main obstacles to overcome this lack of knowledge.

An alternative to perform a rapid identification of species relies on the DNA barcoding approach, based on the sequencing of the mitochondrial gene cytochrome c oxidase subunit I (COX1) and its contrast to previously sequenced specimens with morphological identification and resolved taxonomy (Hebert et al. 2003, Kress and Erickson 2012). The efficacy in the discrimination of fish species, in different stages of development, using COX1 has previously been demonstrated (Ward et al. 2005, Frantine-Silva et al. 2015, Bagley et al. 2019) and the method can be very useful to document the diversity of fishes in megadiverse countries such as Colombia and potentially validate further species still to be described (DoNascimiento et al. 2017).

This contribution to a reference library of DNA barcodes of Colombian freshwater fishes consists of records for 757 specimens deposited in the reference fish collection of the University of Antioquia, collected in the Atrato, Cauca and Magdalena River Basins and morphologically identified to species level, for a total of 96 species of 63 different genera. All specimens have their DNA barcodes made publicly available in the Barcode Of Life System, hereinafter the BOLD System (Ratnasingham and Hebert 2007). The aim of this work is to share and make public the occurrence records and COX1 sequences of specimens present in the reference fish collection at the University of Antioquia and to facilitate the access to available information on Colombian freshwater fishes.

## General description

### Purpose

**Figure 1.**
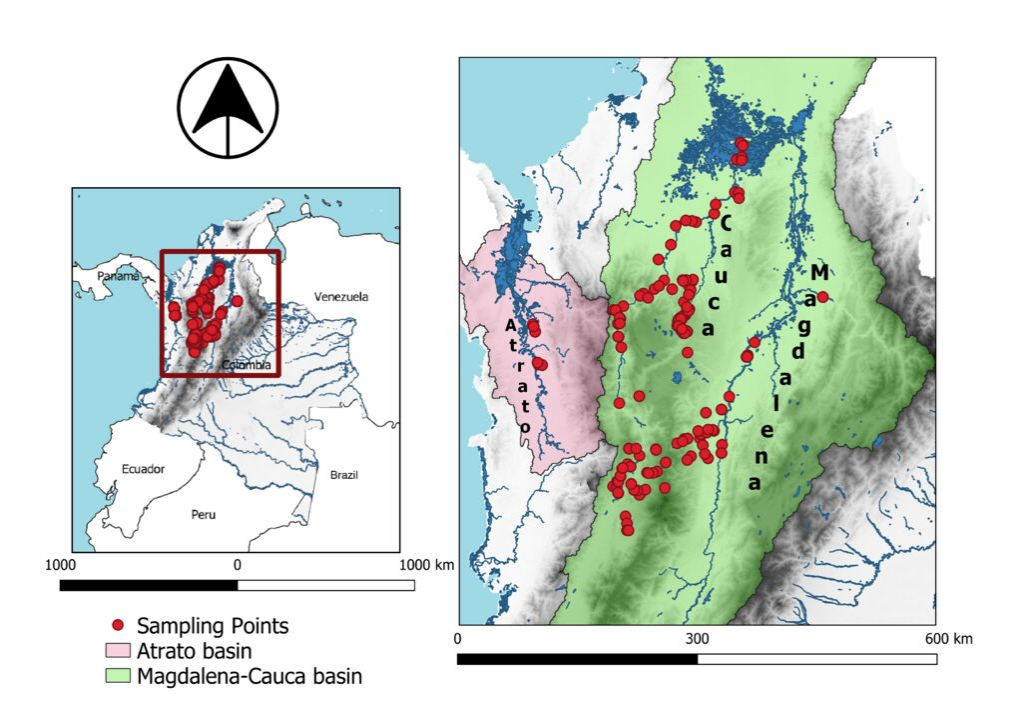
Map of the localities where freshwater fish samples were collected in the Atrato, Cauca and Magdalena River Basins in Colombia.

We aimed to make available a dataset of COX1 sequences of freshwater fish species occurring in the Atrato, Cauca and Magdalena Basins in Colombia (Fig. 1) and, in so doing, provide a molecular tool for the identification of species, future metabarcoding studies, monitoring of the ichthyofauna diversity in these Basins and highlight the value and importance of biological collections.

### Additional information

**Figure 2.**
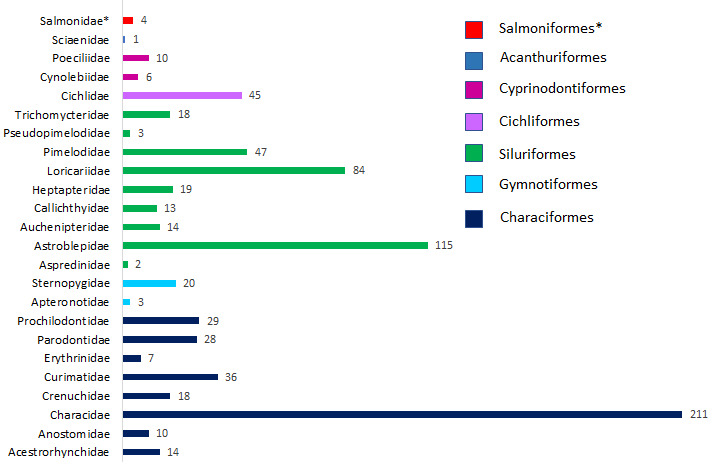
Number of sequences generated per Family. The colour of the bars represents the order and * indicates the family Salmonidae which is exotic to the three River Basins.

A total of 757 specimens from 138 localities were sampled and their COX1 sequences generated. The Cauca, Magdalena and Atrato River Basins are represented by 485 (64%), 223 (29%,) and 49 (7%) sequences, respectively and correspond to 23 families out of a total of 36 reported in DoNascimiento et al. (2017) and the family Salmonidae which is exotic for these three River Basins (Fig. 2). The sequences generated are between 480 and 655 bp long.

## Project description

### Title

"Contribution to a reference library of DNA barcodes for Colombian freshwater fishes."

Refers to the COX1 sequences generated in this study of freshwater fish specimens catalogued in the Ichthyology Collection of the University of Antioquia, which have been reported in the Atrato, Cauca and Magdalena River Basins in Colombia.

### Personnel

Manuela Mejía Estrada (Project developer, Student), Iván D. Soto-Calderón (Project mentor), Luz Fernanda Jiménez Segura (Project coordinator).

### Design description

Freshwater fish specimens were collected in the field using methods defined according to the habitat, including, but not limited to, drift nets, gill nets (1-12 cm, mesh size), hand nets, cast nets (0.5-3 cm mesh size), bottom trawls (0.5-3 cm, mesh size), seine nets, handline and electrofishing. They were also morphologically identified and DNA barcoded.

### Funding

This project was funded by “Empresas Públicas de Medellin, EPM” through the agreement No. CT-2017-001714 with the University of Antioquia.

## Sampling methods

### Study extent

Atrato, Cauca and Magdalena Basins, Colombia.

### Sampling description

The analysed material was collected in 138 different localities. Sampling was conducted between 2010 and 2020 on a wide range of habitats, using the different fishing arts mentioned before. Collected specimens were fixed and stored in alcohol and a portion of muscle or fin was stored in 96% ethanol for downstream molecular analysis. Morphological identification was performed, based on taxonomic keys and descriptions from literature (Suppl. material 1).

DNA was extracted from muscle and/or fin preserved in 96% alcohol using the QIAgen Dneasy Blood & Tissue kit ® (Hilden, Germany), following manufacturer’s protocol. A fragment of approximately 580 bp of the mitochondrial COX1 gene was amplified using the primers FishF1 (5´-TCAACCAACCACAAAGACATTGGCAC-3´) and FISHR1 (5´-TAGACTTCTGGGTGGCCAAAGAATCA-3´) (Ward et al. 2009). A 25 µl PCR cocktail included 2 µl of DNA, 2.5 µl of 10x Taq Buffer, 2.6 µl of 25 mM MgCl_2_, 2.5 µl of 2 mM dNTPs, 1 µl of each primer 2 µM, 0.2 µl (1U) of Taq polymerase and 13.2 µl of water. The thermal profile of the PCR consisted of an initial denaturation step for 5' at 94°C, followed by 35 cycles of 1' at 94°C, 1' at 56°C and 1' at 72°C and then a final pass for 10' at 72°C. The amplification of the single fragment and within the expected size range was verified by 2% agarose electrophoresis. The products were then cleaned with Exonuclease I and Shrimp Alkaline Phosphatase (New England Biolabs, Ipswich, Massachusetts, USA) and sequenced by the standard Sanger method. The forward and reverse sequences were edited and assembled using Geneious Prime (2019) and inspected manually.

### Quality control

The sequences were translated into protein to verify the absence of stop codons and indel events that indicated errors in the sequence or the unintentional amplification of nuclear pseudogenes (numts). The sequences generated are available on the BOLD page (Ratnasingham and Hebert 2007) within the CIUA project.

## Geographic coverage

### Description

Middle to lower portion of the Atrato, Cauca and Magdalena River Basins, continental Colombia (Fig. 1).

### Coordinates

4.53888 and 8.89651 Latitude; -76.81916667 and -73.55097222 Longitude.

## Taxonomic coverage

### Description

This dataset consists of data relating to 757 specimens of freshwater fishes occurring in Colombia; 673 specimens were identified to the species level and 84 to genus. Overall 96 species in 24 families are represented in the dataset (Suppl. material 2). The families Characidae, Astroblepidae and Loricariidae account for 71% of the total collected specimens. The family Sciaenidae is represented by one species with one sequence, whereas seven families (Apteronotidae, Aspredinidae, Callichthyidae, Cynolebiidae, Erythrinidae, Salmonidae (exotic) and Sciaenidae) are represented by a single species with more than one sequence (Suppl. material 3).

## Temporal coverage

**Data range:** 2010-1-01 – 2020-3-01.

## Collection data

### Collection name

Colección de Ictiología de la Universidad de Antioquia CIUA

### Collection identifier

Registro Nacional de Colecciones Biológicas: 168

### Parent collection identifier

CIUA

### Specimen preservation method

ethanol 70%

## Usage licence

### Usage licence

Creative Commons Public Domain Waiver (CC-Zero)

## Data resources

### Data package title

Barcoding CIUA

### Resource link

http://www.boldsystems.org/index.php/Public_SearchTerms?searchMenu=records&query=ciua&taxon=

### Alternative identifiers

dx.doi.org/10.5883/DS-CIUA01

### Number of data sets

1

### Data set 1.

#### Data set name

CIUA01

#### Data format

dwc, xml, fasta

#### Number of columns

18

#### Download URL

http://www.boldsystems.org/index.php/Public_SearchTerms?query=DS-CIUA01

#### Description

The Barcoding CIUA Database: The CIUA01 dataset can be downloaded from the Public Data Portal of BOLD Systems in different formats (data as dwc, xml or tsv and sequences as fasta files). Alternatively, BOLD Systems users can log-in and access the dataset via the Workbench platform of BOLD Systems. All records are also searchable within the BOLD Systems, using the search function of the database.

The Barcoding CIUA will continue sequencing Colombian freshwater fishes for the BOLD Systems database, with the goal of comprehensive coverage.

**Data set 1.** 

Column label	Column description
Project Code	Unique Code for the project.
Process ID	Unique identifier for the sample.
SampleID	ID for the specimen in BOLD Systems Database.
BIN	Barcode Index Number system identifier.
CatalogNum	Number of the record in the collection.
COI-5P Seq. Length	Length of the sequence.
Identification	Current identification of the record.
Institution-Institution Storing	Name of the institution that has physical possession of the voucher specimen.
Museum ID	Unique number of identification for the record at the museum where it is storage.
Phylum	Phylum to which the record belongs.
Class	Class to which the record belongs.
Order	Order to which the record belongs.
Family	Family to which the record belongs.
Genus	Genus to which the record belongs.
Species	Species to which the record belongs.
Country	The full, unabbreviated name of the country where the organism was collected.
Latitude	The geographical latitude (in decimal degrees) of the geographic centre of a location.
Longitude	The geographical longitude (in decimal degrees) of the geographic centre of a location.

## Supplementary Material

BBE30673-B761-56AC-AF79-FD55A316BA68 10.3897/BDJ.10.e65981.suppl1 Supplementary material 1 References used to determine the taxonomy of specimens Data type PDF Brief description List of references used to determine the taxonomy of specimens of CIUA collection File: oo_589552.pdf https://binary.pensoft.net/file/589552 Juan Guillermo Ospina

AB1DFCE5-14F4-5670-9E32-B6E301BC26C2 10.3897/BDJ.10.e65981.suppl2 Supplementary material 2 Barcoding CIUA01-Specimen details Data type Record information, Specimen data. Brief description The file includes information about all records in BOLD Systems for the Barcoding CIUA01 library. It contains collection, location and identification data. File: oo_589963.csv https://binary.pensoft.net/file/589963 Manuela Mejía Estrada, Juliana Herrera, Omer Campo, Marcela Hernandez.

3B5E79A6-5606-59AB-A79D-253E4DB36407 10.3897/BDJ.10.e65981.suppl3 Supplementary material 3 Barcoding CIUA01 Library Data type DNA Sequences, COX1 Sequences. Brief description COX1 sequences in fasta format. Each sequence is identified by the BOLD Sample ID, species name and sequence category, separated by a vertical bar. File: oo_512303.fasta https://binary.pensoft.net/file/512303 Manuela Mejía Estrada, Omer Campo, Marcela Hernandez.
