# Supplementary material for: Contribution to a reference library of DNA barcodes of Colombian freshwater fishes
Source: Biodivers Data J. 2022 Jan 6;10:e65981. doi: 10.3897/BDJ.10.e65981 (PMC8758633; doi:10.3897/BDJ.10.e65981)
Supplement: Supplementary material 1 — References used to determine the taxonomy of specimens [file bdj-10-e65981-s001.pdf]

References used to determine the taxonomy of specimens in the CIUA collection

Agudelo Zamora, H. D., Ortega Lara, A., & Taphorn B., D. C. (2020). Characidium chancoense, a new species of South American darter from the Río Cauca drainage, Colombia (Characiformes: Crenuchidae). *Zootaxa*, *4768*(2), 249–263. https://doi.org/10.11646/zootaxa.4768.2.6

Ardila Rodríguez, C. A. (1994). Peces de agua dulce del departamento del Atlantico, Colombia. *Dugandia*, *5*(1), 3–12.

Armbruster, J. W. (2003). The species of the Hypostomus cochliodon group (Siluriformes: Loricariidae). *Zootaxa*, *249*, 1–60.

Armbruster, J. W. (2005). The loricariid catfish genus Lasiancistrus (Siluriformes) with descriptions of two new species. *Neotropical Ichthyology*, *3*(4), 549–569. https://doi.org/10.1590/s1679-62252005000400013

Ballen, G. A. (2011). *A new species of Chaetostoma T schudi (Siluriformes : L oricariidae) from colombiA wiTh A definiTion of The C. anale species group*. *51*(26), 383–398.

Buitrago Suárez, U. A., & Burr M., B. (2007). Taxonomy of the catfish genus Pseudoplatystoma Bleeker (Siluriformes: Pimelodidae) with recognition of eight species URIEL. *Zootaxa*, *1512*, 1–38. papers3://publication/uuid/08D63A92-6A23-4866-9D35-1970026D41E9

Castellanos Morales, C., Marino Zamudio, L., Guerrero V., L., & Maldonado Ocampo, J. (2011). Peces del departamento de Santander, Colombia. *Revista de La Academia Colombiana de Ciencias Exactas, Físicas y Naturales*, *35*(135), 189–212.

Conde-Saldaña, C. C., Albornoz-Garzón, J. G., García-Melo, J. E., Dergam, J. A., & Villa-Navarro, F. A. (2019). A new species of Pimelodella Eigenmann & Eigenmann, 1888 (Siluriformes: Heptapteridae) from the Sierra Nevada de Santa Marta, Colombia. *Zootaxa*, *4668*(4), 562–574. https://doi.org/10.11646/zootaxa.4668.4.8

Dahl, G. (1955). *An Icthyological Reconnaissance of the Sinu River* (pp. 11–19).

Dahl, G., Meden, F., & Ramos Henao, A. (1963). *El Bocachico contribución al estudio de su biología y de su ambiente*. Departamento de pesca de la coporación autonoma regional de los valles del Magdalena y del Sinu.

Fowler, H. W. (1944). Fresh-Water fishes from Northwestern Colombia. *Proceedings of Academy of Natural Sciences Oh Philadelphia*, *96*, 227–248.

Galvis, G., & Mojica, J. I. (2007). The Magdalena River fresh water fishes and fisheries. *Aquatic Ecosystem Health and Management*, *10*(2), 127–139. https://doi.org/10.1080/14634980701357640

García Alzate, C. A., & Román Valencia, C. (2008). Hyphessobrycon ocasoensis sp. n. (Teleostei, Characidae) una nueva especie para el Alto Cauca, Colombia. *Animal Biodiversity and Conservation*, *31*(2), 11–23.

García alzate, C. A., Román valencia, C., & González, M. I. (2010). Morfogeometría de los peces del género Hyphessobrycon (Characiformes: Characidae), grupo heterorhabdus, en Venezuela.

Rev. Biol. Trop., *58*(3), 801–811.

García Alzate, C. A., Román Valencia, C., & Prada Pedreros, S. (2010). Three new species of Hyphessobrycon group heterorhabdus (teleostei: Characiformes: Characidae) and key to species from the Orinoco river basin. *Caldasia*, *32*(2), 415–433.

Garcia Alzate, C. A., Roman Valencia, C., & Taphorn, D. C. (2008). Revision of the Hyphessobrycon heterorhabdus-group (Teleostei: Characiformes: Characidae), with description of two new species from Venezuela. *Vertebrate Zoology*, *58*(2), 139–157. https://doi.org/10.2471/BLT.10.078964

García Alzate, C. A., Román valencia, C., & Taphorn, D. C. (2008). Two new species of Hyphessobrycon (Pisces : Characiformes : Characidae) from Putumayo River , with keys to the Colombian Hyphessobrycon heterorhabdus – group species. *Brenesia*, *70*, 33–46.

García Alzate, C. A., Román Valencia, C., & Taphorn, D. C. (2010). A new species of Hyphessobrycon (Teleostei: Characiformes: Characidae) from the San Juan River drainage, Pacific versant of Colombia. *Zootaxa*, *2349*, 55–64.

García Melo, J. E., Oliveira, C., Da Costa Silva, G. J., Ochoa Orrego, L. E., Garcia Pereira, L. H., & Maldonado Ocampo, J. A. (2019). Species delimitation of neotropical Characins (Stevardiinae): Implications for taxonomy of complex groups. *PLOS ONE*, *14*(6), 1–22. https://doi.org/10.1371/journal.pone.0216786

Harold, A. S., & Vari, R. P. (1994). Systematics of the trans-Andean species of Creagrutus (Ostariophysi:Characiformes:Characidae). *Smithsonian Contributions to Zoology*, *551*, 1–31. https://doi.org/10.5479/si.00810282.551

Howes, G. (1982). Review of the genus Brycon (Teleostei: Characoidei). *Bulletin of the British Museum of Natural History (Zoology Series)*, *43*(1), 1–47.

Hrbek, T., Seckinger, J., & Meyer, A. (2007). A phylogenetic and biogeographic perspective on the evolution of poeciliid fishes. *Molecular Phylogenetics and Evolution*, *43*(3), 986–998. https://doi.org/10.1016/j.ympev.2006.06.009

Huertas Rodriguez, J. C., Sanín Acevedo, C., & Cataño, A. (2018). Los peces y sus servicios ecosistémicos en la cuenca del río Porce. *Actualidades Biológicas*, *40*(108), 72–84. https://doi.org/10.17533/udea.acbi.v40n108a07

Jiménez-Segura, L., Álvarez, J., Ochoa, L. E., Loaiza, A., Londoño, J. P., Restrepo, D., Aguirre, K., Hernández, A., Correa, J. D., & Jaramillo-Villa, U. (2014). Guía ilustrada de Peces, Cañón del río Porce-Antioquia. In *EPM. Universidad de Antioquia, Herbario Universidad de Antioquia - Medellín, Colombia.* Universidad de Antioquia, Herbario Unibersidad de Antioquia.

Londoño-Burbano, A., & Reis, R. E. (2019). A Taxonomic Revision of Sturisomatichthys Isbrücker and Nijssen, 1979 (Loricariidae: Loricariinae), with Descriptions of Three New Species. *Copeia*, *107*(4), 764–806. https://doi.org/10.1643/CI-19-226

Lujan, N. K., Agudelo-Zamora, H., Taphorn, D. C., Booth, P. N., & López-Fernández, H. (2013). Description of a new, narrowly endemic South American darter (Characiformes: Crenuchidae) from the central Guiana shield highlands of guyana. *Copeia*, *3*, 454–463. https://doi.org/10.1643/CI-12-079

Maldonado Ocampo, J. A., Vari, R. P., & Usma, J. S. (2008). Checklist of the Freshwater Fishes of Colombia. *Biota Colombiana*, *9*(2), 143–237. https://doi.org/10.21068/bc.v9i2.201

Mojica, J. I., Castellanos, C., Sánchez Duarte, P., & Díaz, C. (2006). Peces de la cuenca del río Ranchería, La Guajira, Colombia. *Biota Colombiana*, *7*(1), 129–142. https://doi.org/10.21068/bc.v7i1.168

Nelson, J. S., Grande, T. C., & Wilson, M. V. H. (2016). *Fishes of the world* (5th ed.). Wiley.

Posada. (1909). Los Peces, contribución al estudio de la fauna Colombiana. In *Estudios Científicos* (pp. 285–322).

Ray, C. K., & Armbruster, J. W. (2016). The genera Isorineloricaria and Aphanotorulus (Siluriformes: Loricariidae) with description of a new species. *Zootaxa*, *4072*(5), 501–539. https://doi.org/10.11646/zootaxa.4072.5.1

Restrepo Gómez, A. M., Rangel Medrano, J. D., Márquez, E. J., & Ortega Lara, A. (2020). Two new species of Pseudopimelodus Bleeker, 1858 (Siluriformes: Pseudopimelodidae) from the Magdalena Basin, Colombia. *PeerJ*, *8*. https://doi.org/10.7717/peerj.9723

Ribeiro, D. T. (2006). História evolutiva de espécies do gênero Potamotrygon Garman, 1877 (Potamotrygonidae) na Bacia Amazônica. In *Dissertação INPA*. Universidade Federal do Amazonas.

Román-valencia, C., & Ruiz, R. (2009). Hemibrycon santamartae: a new species from the rancheria river of eastern caribbean colombia (characiformes: characidae). *Rev. Invest. Univ. Quindio*, *19*, 144–150. http://www.uniquindio.edu.co/uniquindio/revistainvestigaciones/adjuntos/pdf/aa34_n1916.pdf

Román valencia, C., Ruiz C., R. I., & Barriga, R. (2006). Una nueva especie de pez del género Hemibrycon (Characiformes: Characidae). *Rev. Biol. Trop.*, *54*(1), 209–218.

Román Valencia, Ceroman, Ruiz C., R. I., & Barriga, R. (2007). Redescripción de Hemibrycon orcesi Böhlke, 1958 y H. polyodon (Gunther, 1864) (Teleostei, Characidae) incluye clave para las especies de Hemibrycon en Ecuador. *Animal Biodiversity and Conservation*, *30*(2), 179–188.

Román Valencia, César, Ruiz C., R., Taphorn, D. C., & Duque, O. (2018). *Guía para la identificación de los peces del río la vieja, alto cauca, Colombia.* (1st ed., Issue November). Universidad del Quíndio.

S. De Lucena, C. A., & Soares, H. G. (2016). Review of species of the Astyanax bimaculatus “caudal peduncle spot” subgroup sensu Garutti & Langeani (Characiformes, Characidae) from the rio la Plata and rio São Francisco drainages and coastal systems of southern Brazil and Uruguay. *Zootaxa*, *4072*(1), 101–125. https://doi.org/10.11646/zootaxa.4072.1.5

Taphorn, D. C., Armbruster, J. W., Villa Navarro, F., & Ray, C. K. (2013). Trans-Andean Ancistrus (Siluriformes: Loricariidae). *Zootaxa*, *3641*(4), 343–370. https://doi.org/10.11646/zootaxa.3641.4.2

Terán, G. E., Benitez, M. F., & Mirande, J. M. (2020). Opening the Trojan horse: Phylogeny of Astyanax, two new genera and resurrection of Psalidodon (Teleostei: Characidae). *Zoological Journal of the Linnean Society*, *190*(4), 1217–1234.

https://doi.org/10.1093/zoolinnean/zlaa019

Urbano Bonilla, A., Ballen, G. A., Herrera R., G. A., Zamudio, J., Herrera Collazos, E. E., DoNascimiento, C., Prada Pedreros, S., & Maldonado Ocampo, J. A. (2018). Fishes of the Cusiana River (Meta River basin, Colombia), with an identification key to its species. *ZooKeys*, *733*, 65–97. https://doi.org/10.3897/zookeys.733.20159

Vanegas Ríos, J. A. (2016). Taxonomic review of the Neotropical genus Gephyrocharax eigenmann, 1912 (Characiformes, Characidae, Stevardiinae). In *Zootaxa* (1st ed., Vol. 4100, Issue 1). Magnolia Press. https://doi.org/10.11646/zootaxa.4100.1.1
